# “Dark” or “Junk”? Functional Roles of Pervasive Transcription in the Human Genome

**DOI:** 10.1093/gbe/evag104

**Published:** 2026-05-14

**Authors:** Haoran Cai

**Affiliations:** Department of Ecology and Evolutionary Biology, University of California, Los Angeles, CA 90095, USA

Highlights editor: Laura A. Katz, Maud Tenaillon

A major debate in genome biology centers on whether the vast, non-protein-coding majority of the human genome consists of nonfunctional “junk” or functional “dark” DNA. This contention is fueled, in part, by the observation of pervasive transcription: while only roughly 2% of the genome encodes proteins, projects like ENCODE reveal that over 75% of it is transcribed into RNA. One view posits that these transcripts represent undiscovered functional noncoding elements, while others argue that this widespread transcription is merely a consequence of background noise—the chance production of many nonfunctional transcripts by a leaky transcriptional machinery (Struhl 2007; van Bakel et al. 2010; Palazzo and Lee 2015).

**Fig. 1.**
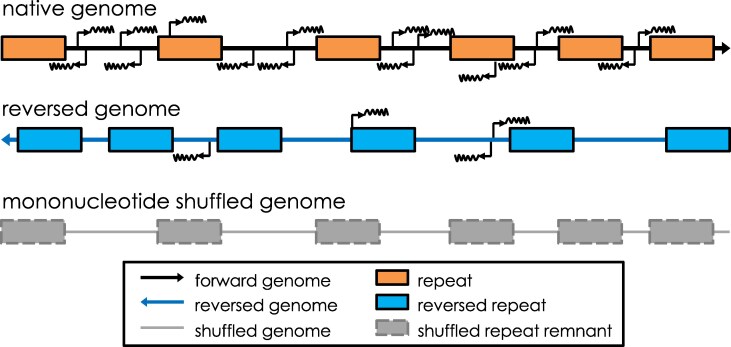
A schematic diagram showing the results from Adey et al. (2026), who used the deep learning model Puffin-D to predict transcription levels in the native and randomized (reversed/shuffled) versions of the human genome. Their results indicate that reversing the whole human genome results in a dramatic reduction in transcription initiation, compared to the native (forward) genome; global mononucleotide shuffling essentially abolishes predicted transcription initiation. Features of the transcription initiation data, such as bias of initiation to non-repeat regions and clustering of initiation sites, are indicated schematically (fuzzy lines); the diagram is not to scale. Figure provided by Austen Ganley.

**Fig. 2.**
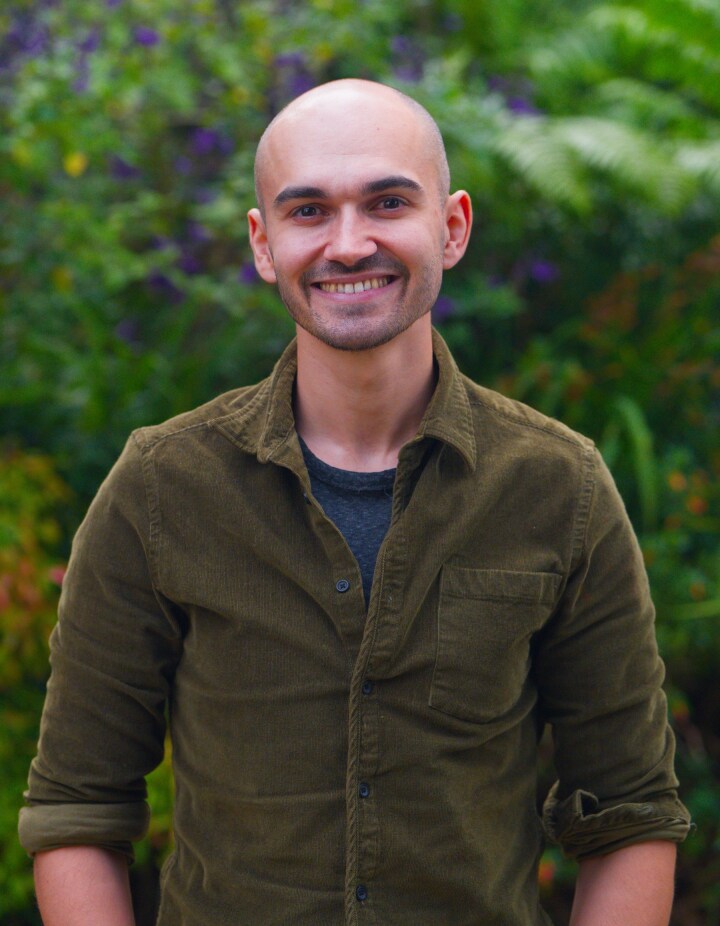
Brett Adey is a final-year PhD student in Dr. Austen Ganley's lab at the University of Auckland. Brett's research explores the relationship between genomic activity and function in the human non-coding genome by applying bioinformatics and machine learning methods to multi-omics data. In his current work, he is investigating whether pervasive transcription is evidence for function by examining a human-Arabidopsis hybrid cell line. More broadly, he is interested in characterizing the principles by which DNA sequence shapes phenotypes.

One could hypothesize that, if transcription levels in random sequences of the genome are comparable to those found in native and evolutionarily shaped genomes, then pervasive transcription might simply reflect background noise rather than functional activity (Eddy 2013). In this month's issue of *Genome Biology and Evolution*, Adey et al. (2026) estimated background transcription by using a deep-learning transcription initiation predictor, Puffin-D, in reversed and shuffled versions of the human genome (Fig. 1). Lead author Brett Adey (Fig. 2)—who conducted the study working collaboratively with Austen Ganley and other colleagues at the University of Auckland, University of Otago, and University of Sydney—explains the backstory behind this ingenious undertaking: “We were less interested in arguments and more interested in evidence, and we liked the random DNA approach for providing evidence on this question,” Adey notes. According to the authors, two recent advances finally made this testable computationally: “The first was the release of a powerful AI model for predicting transcription initiation from the sequence called Puffin-D. The second was the publication of Camellato et al. (2024), which experimentally characterized transcription initiation in two related, large pieces of random DNA. Their data enabled us to test whether Puffin-D can predict transcription initiation in “random” DNA.”

To evaluate baseline transcription genome-wide, Adey et al. (2026) created randomized human genomes *in silico* by reversing or shuffling sequences, destroying the encoded biological information while preserving varying degrees of local sequence composition (Fig. 1). Upon applying Puffin-D to these altered sequences, researchers noted that while some background transcription exists, predicted initiation levels of transcription remained sparse. In fact, these predicted transcription initiation levels were at least four times lower than the levels detected in the native human genome. Such a significant difference suggests that most transcription in humans is not simply the result of stochastic background noise.

If background noise cannot explain this excess transcription, could transposable elements (TEs) be the driver? TEs comprise a large portion of the human genome, and have been proposed to contribute substantially to pervasive transcription. To test this, the authors computationally partitioned the genome and randomized only the interspersed repeat regions—predominantly composed of TEs—and compared them with the non-repeat regions. The results were telling: perturbing non-repetitive DNA reduced its transcription rate, whereas shuffling repetitive DNA resulted in transcription levels that are similar to the repeat regions in the native human genome.

Adey interprets these contrasting patterns as an indication of how information is stored in different parts of the genome: “We think the results are compatible with the non-repeat parts of the native genome containing initiation sites that are specifically encoded, whereas the transcription initiation seen in the repeats is mostly background.” Therefore, perturbing the repeats does not result in dramatic drops in transcription levels because the initiation there is already at baseline.

Surprisingly, unlike shuffling the repetitive DNA, reversing them resulted in much higher rates of transcription initiation in these repeat regions. Adey speculates this result could relate to transposon insertion mechanisms. “Target-primed reverse transcription leaves an A/T-rich region around the 3′ end of the transposon. When reversed, this would give an A/T-rich region at the 5′ end of the transposon, and such A/T-rich regions may be enriched for chance occurrences of transcription initiation sites.”

Given that pervasive transcription exceeds background noise and cannot be fully explained by TEs, the authors propose two explanations for the excess: selection or mutational processes. Specifically, excess transcription might provide selective benefits, either through the production of functional noncoding RNAs or due to the physical act of transcription itself. It is possible that the process of transcribing DNA—rather than the resulting RNA transcript—has been selected for, for example, by displacing DNA-bound proteins to aid in gene regulation (Kaikkonen and Adelman 2018). Alternatively, mutational processes might naturally skew the genome, leading to overrepresentation of sequences capable of transcription initiation.

To experimentally or computationally distinguish between these evolutionary origins, Ganley emphasizes that researchers first need “a fairly precise understanding of the sequences that encode transcription initiation sites. Without knowing this, it would be very hard to determine whether mutation biases are likely to produce sequences enriched for such initiation sites.” With recent progress in the field, this could soon be evaluated computationally or experimentally via large-scale promoter activity assays. However, Ganley cautions that such tests can only offer a statistical bound for how much transcription initiation could be attributed to mutational bias: “it is likely to be very difficult to discriminate between selection and bias for any given transcript with these tests.” In any case, and regardless of the evolutionary mechanisms underlying this process, Adey et al. (2026) suggest that pervasive transcription is not merely a consequence of background noise.

*Want to learn more?* Check out these other articles on the “dark” or “junk DNA” published in *Genome Biology and Evolution*:

- Dan Graur, An Upper Limit on the Functional Fraction of the Human Genome, *Genome Biology and Evolution*, Volume 9, Issue 7, July 2017, Pages 1880–1885, https://doi.org/10.1093/gbe/evx121.
- Dan Graur, Yichen Zheng, Nicolas Price, Ricardo B. R. Azevedo, Rebecca A. Zufall, Eran Elhaik, On the Immortality of Television Sets: “Function” in the Human Genome According to the Evolution-Free Gospel of ENCODE, *Genome Biology and Evolution*, Volume 5, Issue 3, March 2013, Pages 578–590, https://doi.org/10.1093/gbe/evt028.
- Nelson J.R. Fagundes, Rafael Bisso-Machado, Pedro I.C.C. Figueiredo, Maikel Varal, André L.S. Zani, What We Talk About When We Talk About “Junk DNA”, *Genome Biology and Evolution*, Volume 14, Issue 5, May 2022, evac055, https://doi.org/10.1093/gbe/evac055.
- Brent M. Robicheau, Edward Susko, Amye M. Harrigan, Marlene Snyder, Ribosomal RNA Genes Contribute to the Formation of Pseudogenes and Junk DNA in the Human Genome, *Genome Biology and Evolution*, Volume 9, Issue 2, February 2017, Pages 380–397, https://doi.org/10.1093/gbe/evw307.
